# PI3K/Akt/mTOR Signaling Pathway: Role in Esophageal Squamous Cell Carcinoma, Regulatory Mechanisms and Opportunities for Targeted Therapy

**DOI:** 10.3389/fonc.2022.852383

**Published:** 2022-03-22

**Authors:** Qian Luo, Ruijuan Du, Wenting Liu, Guojing Huang, Zigang Dong, Xiang Li

**Affiliations:** ^1^ Department of Pathophysiology, School of Basic Medical Sciences, Zhengzhou University, Zhengzhou, China; ^2^ China-US (Henan) Hormel Cancer Institute, Zhengzhou, China; ^3^ Henan Provincial Cooperative Innovation Center for Cancer Chemoprevention, Zhengzhou University, Zhengzhou, China; ^4^ State Key Laboratory of Esophageal Cancer Prevention and Treatment, Zhengzhou University, Zhengzhou, China

**Keywords:** PI3K/Akt/mTOR pathway, ESCC, inhibitor, drug resistance, mutation

## Abstract

Esophageal squamous cell carcinoma (ESCC), is the most common type of esophageal cancer worldwide, mainly occurring in the Asian esophageal cancer belt, including northern China, Iran, and parts of Africa. Phosphatidlinositol 3-kinase (PI3K)/protein kinase B (Akt)/mammalian target of rapamycin (mTOR) signaling pathway is one of the most important cellular signaling pathways, which plays a crucial role in the regulation of cell growth, differentiation, migration, metabolism and proliferation. In addition, mutations in some molecules of PI3K/Akt/mTOR pathway are closely associated with survival and prognosis in ESCC patients. A large number of studies have found that there are many molecules in ESCC that can regulate the PI3K/Akt/mTOR pathway. Overexpression of these molecules often causes aberrant activation of PI3K/Akt/mTOR pathway. Currently, several effective PI3K/Akt/mTOR pathway inhibitors have been developed, which can play anticancer roles either alone or in combination with other inhibitors. This review mainly introduces the general situation of ESCC, the composition and function of PI3K/Akt/mTOR pathway, and regulatory factors that interact with PI3K/Akt/mTOR signaling pathway. Meanwhile, mutations and inhibitors of PI3K/Akt/mTOR pathway in ESCC are also elucidated.

## 1 Introduction

Esophageal cancer is the sixth leading cause of cancer death worldwide. According to statistics, there are more than 604,000 new cases of esophageal cancer diagnosed in 2020, of which about 544,000 died from it. In developed countries, the 5-year survival rate of ESCC is less than 20%, and in many developing countries, the 5-year survival rate is less than 5% (1). Esophageal cancer is mainly divided into esophageal squamous cell carcinoma (ESCC) and esophageal adenocarcinoma (EAC), among which ESCC is the most common histological type in the “Asian esophageal carcinoma belt”, including Iran, Kazakhstan and northern China. Risk factors for ESCC mainly include gender, race, smoking, alcohol, diet, nutrition and gene alteration, etc. (2).

The phosphatidlinositol 3-kinase (PI3K)/protein kinase B (Akt)/mammalian target of rapamycin (mTOR) signaling pathway is one of the most vital and most frequently altered signaling pathways in organisms. Studies have pointed out that the major components dysregulation of this signaling pathway led to aberrant activation of the downstream pathways, which ultimately promoted occurrence of cancer.

PI3Ks, members of the lipid kinase family, are usually activated by receptor tyrosine kinases (RTK) and G-protein coupled receptors (GPCR). Moreover, phosphatidylinositol (PI) is a membrane phospholipid, which occupies a small proportion in the composition of cell membrane. The inositol ring of PI can be phosphorylated at several sites, especially 4 and 5. These two sites are phosphorylated by various kinases, leading to the formation of PIP2 (phosphatidylinositol 4, 5 -biphosphate) (3). PI3Ks can be divided into three categories, namely class I, class II and class III. The most widely studied class I PI3Ks is a heterodimer, composed of a catalytic subunit (p110) and a regulatory subunit (p85). Class II PI3Ks include PI3K-Cα, PI3K-Cβ and PI3K-Cγ; Class III PI3Ks PIK3C3, also known as vacuolar protein sorting 34 (VPS34). Upon receiving signals from RTKs and GPCRs, the p85 regulatory subunit of PI3K is recruited to the adjacent plasma membrane, where the p110 subunit binds to the p85 subunit to convert the substrate phosphatidylinositol 2 phosphate, PtIns (4, 5)P2(PIP2) into PtIns (3–5)P3(PIP3) for subsequent reactions (4).

Akt is a serine/threonine kinase and a key regulator of the PI3K/Akt/mTOR signaling pathway. There are three subtypes of Akt (Akt1/PKBα, Akt2/PKBβ, and Akt3/PKBγ), which are encoded by different genes and differ greatly in their distribution. PIP3 binds to the N-terminal pH domain of protein kinase B (PKB, Akt) to transfer Akt from the cytoplasm to the cell membrane. Akt is activated by phosphorylation of the threonine phosphorylation site (Thr308) and serine phosphorylation site (Ser473) with the help of phosphoinositide-dependent protein kinase-1 (PDK1) and phosphoinositol-dependent protein kinase-2 (PDK2). Most interestingly, Akt is also activated by mTOR feedback, this activation of Akt is regulated by the mammalian target of rapamycin complex 2 (mTORC2), and it does not require PDK1 participation (5). Phosphatase and tensin homolog deleted on chromosome ten (PTEN) is a classical tumor suppressor involved in the regulation of the PI3K/Akt pathway. Its main function is to hydrolyze PIP3 into PIP2 and prevent Akt activation.

mTOR is a serine/threonine kinase that typically assembled into a variety of complexes, such as mammalian target of rapamycin complex 1(mTORC1) and mammalian target of rapamycin complex 2(mTORC2). In addition to its core protein component, mTOR, mTORC1 also includes raptor (mTOR regulatory related protein), mLST8 (GβL), PRAS40 (proline-rich Akt substrate) and DEPTOR (protein containing the DEP domain).Interestingly, mTORC2 contains the same mTOR, DEPTOR and mLST8 as mTORC1, and it also contains own unique components, PROTOR, rictor and mSIN1. Activated Akt can activate its substrate mTOR through direct and indirect pathways, such as direct phosphorylation of mTOR, or through inactivation of tuberous sclerosis complex 2 (TSC2), and then enhance activation of mTOR (6).

## 2 Mutations of PI3K/Akt/mTOR Pathway in ESCC

There are many abnormal mutations in PI3K/Akt/mTOR pathway, such as PIK3CA and Akt subtype mutations, which can activate PI3K/Akt/mTOR pathway and affect the occurrence of ESCC. Among them, mutations in the PIK3CA gene encoding p110α are common in ESCC (3). A study demonstrated that PIK3CA mutations were detected in exon 9 or exon 20 in 46 (21%) of 219 cases of ESCC (7). Moreover, Chang et al. comprehensively analyzed the genomic changes of 94 ESCC tumor samples through whole genome sequencing, and found that the amplification rate of PIK3CA in these samples was 38.3%(36/94). However, both PIK3CA mutation and PIK3CA amplification were present in only 2 samples. Surprisingly, the study also analyzed the mutant spectrum of HNSCC, LUSCC and EAC, and found that the mutations of ESCC were similar to HNSCC and LUSCC, but quite different from EAC. This means that mutations in cancers of the same tissue are largely similar, regardless of whether they are the same organ (8). Wang and his colleagues also showed that this is the case. When they analyzed the epithelial cell genomes of advanced ESCC and EAC, they found that the genomes of ESCC and EAC were different. The mutation rate of PIK3CA in 71 ESCC cases was up to 24%, mainly including amplification, base substitution and short indels. Likewise, PTEN mutation rate could be up to 11%, including truncated mutations, base substitutions and short indels. However, the mutation rate of PIK3CA in EAC was only 10%, and that of PTEN was 4%. It was worth noting that Akt1 was only slightly amplified in EAC (9). Moreover, a study by Zhang et al. found that PIK3CA was the most frequently altered gene in the PI3K/Akt/mTOR pathway, with about 17% mutation rate. Hot spot mutation of PIK3CA (c.1624G&gt; A [p.Glu542Lys] and c. 1633 g & gt; A [P.Glu545lys]) was enriched in ESCC with the characteristics of APOBEC (10). In addition to PIK3CA mutations, single nucleotide polymorphisms (SNPs) of several genes in the Akt signaling pathway are also associated with susceptibility to ESCC. Zhu et al. showed that there were significant gene-gene interactions among the three Akt1 SNPs. Akt1 rs2294750 alone or in combination with two other Akt1 SNPs (rs2494752, rs10138277) can jointly combat ESCC, especially in women and non-alcoholic ESCC patients (11). Michelle A.T et al. identified mutations in Akt1, Akt2, PIK3CA, PTEN, and FRAP1 in 174 resectable adenocarcinoma and 36 squamous cell carcinoma patients. Additionally, this study demonstrated a significant association between these common genetic variations and clinical outcomes (12). In 1116 patients with ESCC and 1117 non-cancer controls, Zhu et al. found that three SNPs of mTOR were significantly associated with increased risk of ESCC, highlighting the influence of gene-gene and gene-environment interactions (13). Similarly, Hongping Yu et al. identified 8 functional SNPs of mTORC1 that individually or collectively contribute to ESCC risk in 1126 patients with ESCC and 1131 non-cancer controls (14). Yang et al. sequenced the genomes of 24 ESCC specimens and found that the probability of mTOR gene alteration was 25% (6/24). Of the 115 genes detected, only Akt2 and PIK3CA amplification were found, and the frequency of amplification was 4.2% (1/24). These genetic alterations provide potential targets for future therapies of ESCC (15).

## 3 Role of PI3K/Akt/mTOR Signaling Pathway in ESCC

PI3K/Akt/mTOR pathway is essential for the growth and development of ESCC cells. It is involved in multiple stages of cell growth and differentiation, in the meantime, related to many aspects such as cell metastasis, proliferation and apoptosis. In order to understand the specific role of PI3K/Akt/mTOR pathway in ESCC, Lee et al. knocked down mTOR, raptor, rictor and applied mTOR inhibitors respectively. Knocking down raptor and rictor in TE8 cells significantly reduced the proliferation of the cells compared with non-silencing siRNA (16). Rapamycin, an inhibitor of mTOR. It can also inhibit proliferation of ESCC cells, but to a lesser extent than mTOR knockdown. In addition, knockdown of mTOR, raptor, and rictor induced G1 phase cell arrest. Interestingly, both downregulation of raptor or administration of rapamycin induced mild apoptosis. However, downregulation of mTOR and rictor were not associated with apoptosis (16). Hou et al. conducted similar studies and found that siRNA could significantly down-regulate the level of mTOR and its downstream factors, p-p70S6K and p-4E-BP1, promoting their non-phosphorylation (17). Another study showed that siRNA inhibited the expression of Akt in TE-1 and TE-5 cells, leading to a decrease in MDM2 levels. MDM2 has been shown to form a tight complex with wild-type p53. Hence, the function of wild-type p53 can be inhibited by changing the level of MDM2 (18).

In addition to the above effects, the PI3K/Akt/mTOR pathway has been reported to be closely related to the prognosis of ESCC. A study conducted by Wu et al. showed that mTOR, p-mTOR and p70S6K1 were prognostic factors for progression-free survival (PFS). The expression of mTOR, p-mTOR, p70S6K1 and PTEN were associated with lymph node metastasis and late TNM staging of ESCC (19). Another study showed a positive correlation between periostin and mTOR in locally advanced ESCC, which are independent risk factors for overall survival (OS) and PFS in ESCC patients (20). Moreover, Lee et al. demonstrated that p-mTOR/mTOR is inversely proportional to disease-specific survival, meanwhile it is a more powerful prognostic factor for ESCC than p-mTOR (16). Apart from mTOR, PI3Ks has also been reported to affect the prognosis of ESCC. The expression of PI3K was positively correlated with the degree of clinical stage, depth of invasion and differentiation. But PI3K can only be used as a reference for poor prognosis of ESCC, rather than an independent prognostic indicator (21). One study showed that the level of p-Akt was the only independent factor affecting the prognosis of ESCC patients with chemotherapy. The level of p-Akt increased significantly after chemotherapy, while p-mTOR did not change. It was also pointed out that p-Akt was correlated with the depth of tumor invasion before chemotherapy, while it was not correlated with any clinicopathological parameters after chemotherapy (22). However, Shan et al. believed that p-Akt was associated with lymph node metastasis and tumor differentiation degree, and cumulative survival was significantly higher in p-Akt negative patients than in p-Akt positive patients (23). Additionally, a study have showed that both expression level of RNF2 and p-Akt can affect the OS of patients with ESCC, and RNF2 positive/p-Akt-positive ratio was an independent prognostic factor for ESCC (24).

## 4 Molecules Regulating PI3K/Akt/mTOR Pathway in ESCC

**Figure 1:**
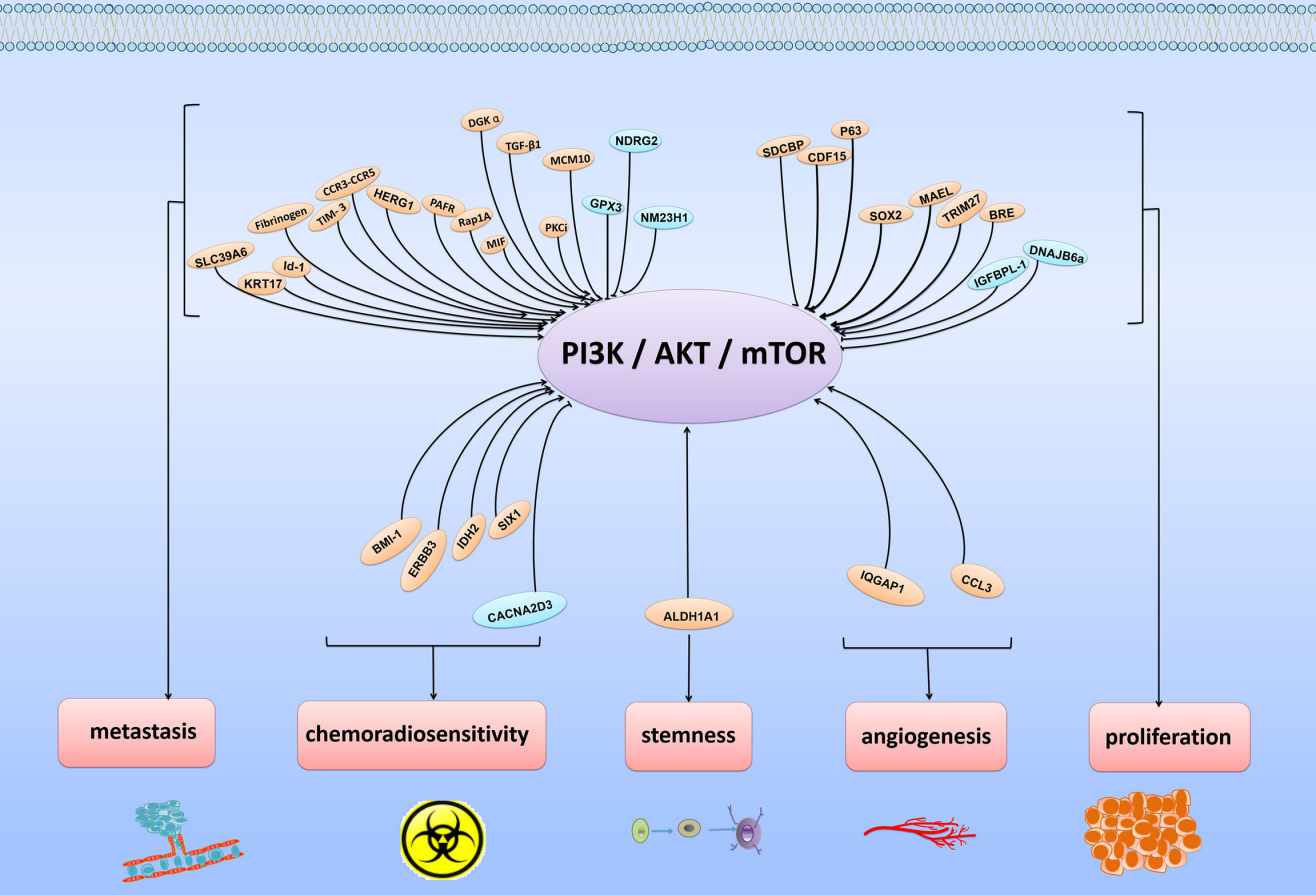
Various regulatory molecules of PI3K/Akt/mTOR signaling pathway and physiological functions of these molecules. The PI3K/Akt/mTOR signaling pathway is usually regulated by various signaling molecules. By targeting major molecules in the PI3K/Akt/mTOR signaling pathway, these molecules play positive or negative roles in regulating cancer proliferation, metastasis, angiogenesis, stemness and chemoradiosensitivity.

The PI3K/Akt/mTOR pathway is always activated and plays critical roles in the development and progression of ESCC. As shown in **Figure 1**, in ESCC, many molecules can participate in regulating the activity of this pathway, finally facilitating cell proliferation, metastasis and chemoradiosensitivity.

### 4.1 Molecules Regulating ESCC Proliferation

#### 4.1.1 Positive Regulators of PI3K/Akt/mTOR Pathway on ESCC Proliferation

SDCBP was a crucial promoter of tumor proliferation. Meanwhile, it is also a downstream factor of AURKA. PDZ2 domain of SDCBP can directly bind with EGFR, thereby activating EGFR and PI3K/Akt pathway (25). CDF15, P63 and SOX2 significantly enhanced proliferation of ESCC cells that was mediated, at least in part, through activation of Akt pathway. Notably, overexpression of P63 observably increased the level of p-Akt without affecting Akt (26–28). Moreover, it has been reported that ectopic expression of MAEL promoted tumor cell growth. The mechanism was that MAEL upregulated IL-8 by activating the Akt1/RelA signaling pathway (29). BRE is a stress-responsive gene, and its overexpression significantly promoted the proliferation of ESCC cells. One study indicated that BRE could negatively regulate the expression of PTEN to activate the Akt pathway and promote the occurrence and development of tumors (30). It is interesting to note that TRIM27 was a pro-proliferation factor in ESCC and it could also interact with PTEN to promote poly-ubiquitination. Thus, the activity of PI3K/Akt pathway was increased (31).

#### 4.1.2 Negative Regulators of PI3K/Akt/mTOR Pathway on ESCC Proliferation

In the process of tumor development, there are also many tumor suppressors, which inhibit the proliferation of cells through inactivating PI3K/Akt/mTOR pathway. IGFBPL-1 belonged to IGFBP family and was a tumor suppressor in ESCC. It inhibited proliferation and induced apoptosis in esophageal cancer cells by attenuating PI3K/Akt signaling pathway (32). Additionally, it has been reported that DNAJB6a suppressed ESCC cell proliferation by inhibiting Akt signaling and the activity of functional protein phosphatase 2A (PP2A). It’s worth noting that PP2A was required for DNAJB6a to regulate Akt signaling (33).

### 4.2 Molecules Regulating ESCC Metastasis

#### 4.2.1 Positive Regulators of PI3K/Akt/mTOR Pathway on ESCC Metastasis

Epithelial-mesenchymal transition (EMT), an embryonic program, loosens cell-cell adhesion complexes and enhances cell migration and invasion. In cancer, EMT is associated with tumor initiation, invasion, metastasis, and resistance to treatment (34). Id-1 and HERG1 can regulate EMT, at least in part by activating the PI3K/Akt pathway to promote migration and invasion of ESCC cells. Their mechanisms are that HERG1 participates PI3K/Akt pathway by targeting TXDC5, while Id-1 can directly affect PI3K/Akt pathway (35, 36). Furthermore, we found that many proteins have similar effects. For example, Rap1A and KRT17-induced EMT are driven by the Akt signaling in ESCC. Their overexpression could accelerate cell metastasis by enhancing cell migration and invasion (37, 38). Moreover, TGF-β1 mediated EMT *via* PTEN/PI3K pathway (39). TIM-3 induced EMT is driven by Akt/GSK-3β/snail signaling pathway (40). Additionally, fibrinogen and MIF can mediate EMT *via* p-AKT/p-mTOR and Akt/GSK-3β/β-catenin pathway, respectively (41, 42). In addition to above molecules, there are other molecules that promote metastasis through this pathway, such as SLC39A6, a member of ZRT, IRT-like protein (ZIP) family. At the same time, SLC39A6 is a zinc importer whose roles on promoting migration and invasion of ESCC cells might be related to intracellular zinc accumulation. The underlying mechanism is that SLC39A6 and cellular zinc could active the PI3K/Akt and MAPK/ERK signaling pathways, thus promoting the occurrence and development of ESCC (43). PKCi and PAFR can promote metastasis of esophageal cancer by indirectly regulating the PI3K/Akt pathway. This is because PKCi and PAFR can directly target SKP2 and FAK, thereby affecting the PI3K/Akt signaling pathway (44, 45). On the contrary, MCM10 and CCR3-CCR5 axis induce migration and invasion of ESCC cells through direct regulation of Akt and PI3K/Akt pathways, respectively (46, 47). Not only that, DGKα can also stimulate metastasis of ESCC. The mechanism is that DGKα actives Akt/NF-κB signaling pathway by directly binding with the FERM domain of FAK *via* its catalytic domain. Moreover, DGKα-mediated phosphatidic acid (PA) production can inhibit the activity of CAMP/PTEN and improve the Akt activation (48).

#### 4.2.2 Negative Regulators of PI3K/Akt/mTOR Pathway on ESCC Metastasis

In addition to the above-mentioned molecules that can positively regulate PI3K/Akt/mTOR pathway to promote EMT, there are also some negative regulatory molecules. For instance, upregulation of NDRG2 inhibited Akt/XIAP signaling pathway and the expression of EMT-related proteins, thereby suppressing the migration, invasion and tumor formation of esophageal cancer cells (49). More interestingly, Nm23H1 can also suppress cell invasion and EMT by negatively regulating Akt activation (50). GPX3 is another negative regulator of FAK/Akt signaling pathway. GPX3 can inhibit expression of MMP9, a substance that contributes to invasion, through deactivating FAK/Akt pathway and suppressing tumor metastasis (51).

### 4.3 Molecules That Regulate Chemoradiotherapy Sensitivity

Chemotherapy and radiotherapy are two common cancer treatments in ESCC. But they are often limited by intrinsic factors of tumor cells. Some proteins can affect the radioresistance and chemoresistance of tumor cells by regulating the PI3K/Akt/mTOR pathway.

#### 4.3.1 Positive Regulators of PI3K/Akt/mTOR Pathway to Increase Chemoradiotherapy Sensitivity

BMI-1, the core component of PcG, is abnormally expressed in various kinds of cancers, including ESCC. BMI-1 regulated the expression of proteins related to DNA damage repair, such as γH2AX, MDC1 and 53BP1. Moreover, in ESCC,BMI-1 could also involve in the regulation of radiosensitivity. Downregulation of BMI-1 significantly decreased the proportion of G2/M phase cells by inhibiting the PI3K/Akt/mTOR pathway, and reduced the chance of DNA damage repair, and ultimately increased radiosensitivity (52). ERBB3 is a gene that has been reported to be associated with the PI3K/Akt signaling pathway. One study revealed that HOXC10 could directly bind with Ku70 and the promoter region of ERBB3 to facilitate DNA damage repair and upregulate ERBB3 transcription, thereby activating the PI3K/Akt signaling pathway and inducing resistance to chemoradiotherapy (53). Moreover, IC50 value of cisplatin was positively connected with HOXC10 expression, suggesting that HOXC10 was involved in chemotherapy resistance (53). In addition, IDH2 and SIX1 have also been reported to be involved in the regulation of radiosensitivity. The increased radiosensitivity induced by IDH2 knockdown that depends on the decreased phosphorylation of Akt. Likewise, overexpression of SIX1 induced radioresistance through activation of the Akt signaling pathway (54, 55).

#### 4.3.2 Negative Regulators of PI3K/Akt/mTOR Pathway to Induce Chemoradiotherapy Sensitivity

Cisplatin is a well-known chemotherapeutic drug. It has been used to treat numerous cancers such as lung, ovarian, and testicular cancers. However, it also has drug resistance and many undesirable side effects. CACNA2D3 is a gene that is located at 3p29.1 on the short arm of chromosome 3. It has been found to have potential anticancer function in many kinds of tumors. IC50 value of cisplatin was negatively correlated with CACNA2D3 expression in ESCC cells, in the meantime, CACNA2D3 can enhance cisplatin sensitivity by inhibiting the PI3K/Akt pathway (56).

### 4.4 Other Molecules

Both IQGAP1 and CCL3 could promote angiogenesis in ESCC, and their mechanisms were similar. IQGAP1 facilitated tumor angiogenesis by targeting the VEGF-VEGFR2 signaling pathway mediated *via* Akt and ERK (57). CCL3-CCR5 axis upregulated the level of VEGF-A through activating PI3K/Akt and MEK/ERK signaling pathway, thereby promoting ESCC angiogenesis (47). Moreover, ALDH1A1 is a marker of cancer stem-like cells. One study indicated that overexpression of ALDH1A1 could maintain the cancer stem-like cells characteristics of ESCC and enhance the levels of Akt1, p-Akt (T308), p-Akt(S473) and β-catenin by activating the Akt signal pathway and binding with β-catenin (58).

## 5 Targeting the PI3K/Akt/mTOR Pathway in ESCC Therapeutics

**Figure 2:**
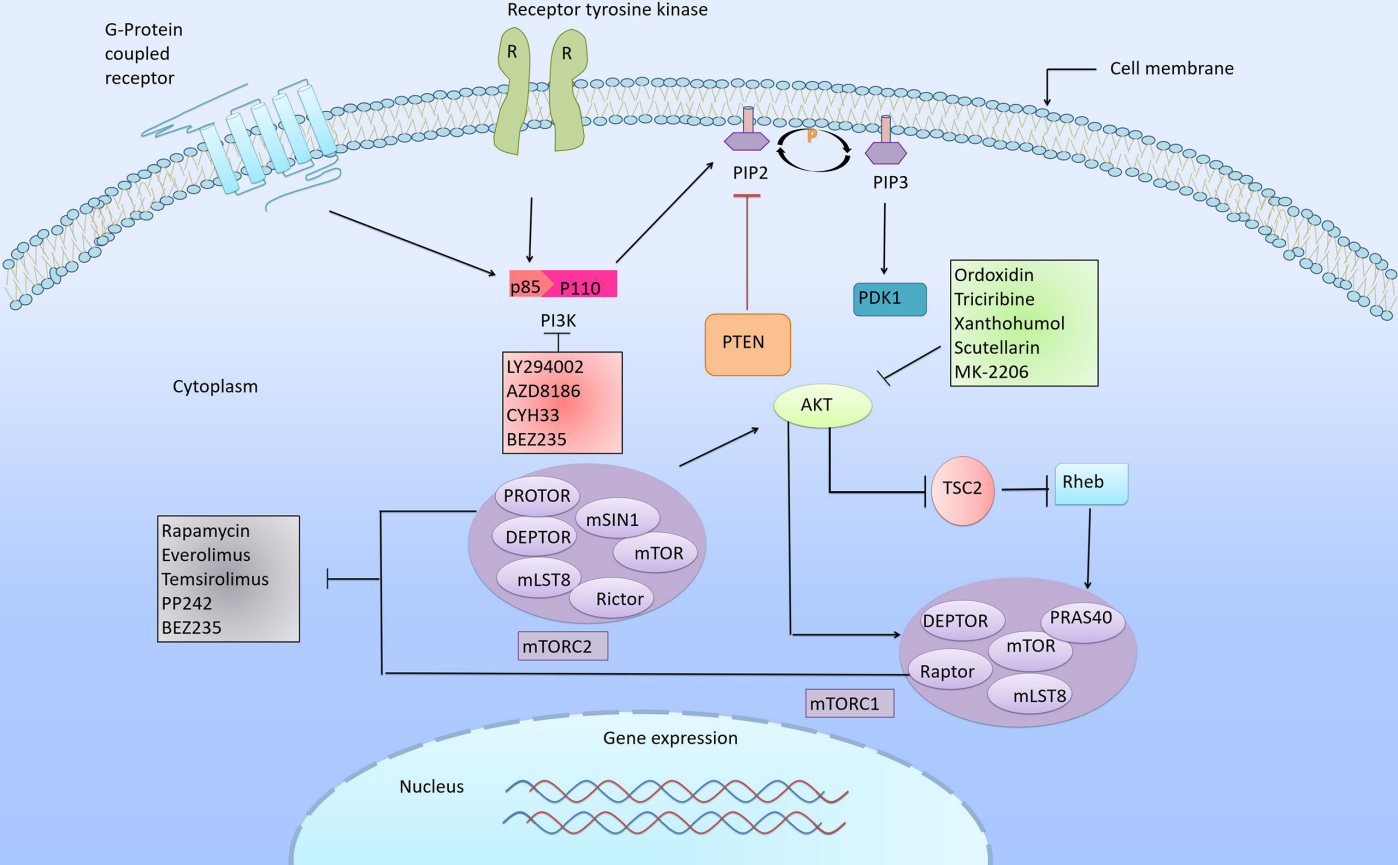
PI3K/Akt/mTOR signaling cascade and its corresponding inhibitors. The PI3K/Akt/mTOR pathway is one of the most frequently altered pathways in cancer, and plays an important role in the regulation of cell growth, differentiation, proliferation, apoptosis and metastasis. Abnormal signal transduction in this pathway is closely related to the progression of cancer. Arrows indicate activation and bars indicate inhibition. The various inhibitors targeting PI3K/Akt/mTOR pathway proven to have inhibitory effects in ESCC.

Aberrant activations of the PI3K/Akt/mTOR signaling pathway are common in human cancers, including ESCC. It has been reported that the abnormal activations of this pathway were closely related to the dysregulated expression of PI3K, Akt and mTOR, which lay foundations for targeted therapy. In this review, three types of inhibitors in ESCC will be introduced, namely Akt inhibitors, PI3K inhibitors and mTOR inhibitors (**Figure 2** and **Supplementary Table S1**).

### 5.1 Akt Inhibitors

Oridonin, a diterpenoid compound extracted from Rabdosia rubescens, has been used to treat a variety of diseases, with cancer being the most notable one. A study conducted by Song et al. found that oridonin can directly interact with Akt1/2, inhibit the activity of Akt1/2 kinase and competitively bind with ATP. Thus, the proliferation of ESCC cells was inhibited, apoptosis and G2/M phase arrest were induced (59). Another compound, triciribine (TCN), was a highly effective radiation sensitizer for ESCC cells *in vitro* and *in vivo*. In ESCC cells and xenograft models, TCN enhanced the radiation sensitivity of ESCC cells by inhibiting hypoxia-induced Akt and HIF-1α expression (60). Xanthohumol is an ATP-competitive Akt kinase inhibitor. It was reported that xanthohumol can induce apoptosis and G1 phase cell arrest. Furthermore, it can suppress phosphorylation of GSK3β, mTOR and ribosomal protein S6, which are downstream targets of Akt, by directly inhibiting Akt 1/2 (61). The scutellarin extracted from scutellaria barbata is another ATP-competitive Akt inhibitor. A recent study has demonstrated that scutellarin can induce G2 cell cycle arrest and show anticancer effects *in vitro* and *in vivo*. Furthermore, it suppressed GSK3-β phosphorylation by directly targeting Akt1 and Akt2 (62). Unlike ATP-competitive inhibitors, allosteric Akt inhibitors including MK-2206 do not cause hyperphosphorylation of Akt at Ser473/Thr308. *In vitro* phenotypic and xenograft mouse models of ESCC, the combination of MK-2206 and BEZ235 was found to be more effective than monotherapy (63, 64).

### 5.2 PI3K Inhibitors

BEZ235 is an ATP-competitive dual pan-PI3K and mTORC1/mTORC2 inhibitor. Because it can target more than one molecule at the same time, it shows a brighter future in cancer therapy. As mentioned earlier, BEZ235 can be used in combination with MK-2206 to inhibit the progression of ESCC. In addition, it also worked in combination with histone deacetylase inhibitor, trichostatin A (TSA). Wu et al. revealed that co-treatment with BEZ235 and TSA improved effects of single drug on cell proliferation, apoptosis and autophagy in ESCC. Moreover, the combination treatment significantly suppressed the phosphorylation of mTOR, Akt, and p70S6K (65). LY294002 is described as the first-generation pan-PI3K inhibitor. Some studies have shown that LY294002 inhibited the proliferation of ESCC cells not only through decreasing the levels of PI3Kp85α, p-Akt (Thr308) and p-p70S6K, but also increasing the expressions of p-Akt (Ser473) and PRAS40 (Thr246) (66). Another study demonstrated that knockdown rictor was capable of inhibiting LY294002-induced Akt compensatory activation, as well as synergically suppressing PI3Kp85α, p-Akt (Thr308) and p-P70S6K. Therefore, the sensitivity of ESCC cells to LY294002 was effectively improved (67). Additionally, AZD8186 is a specific inhibitor of PI3Kβ and PI3Kδ, which was currently in clinical trials and has shown strong anticancer effects. A recent study found that combining DTX and AZD8186, with a disulfide cross-linked micelle (DCM) -based approach, significantly increased the phosphorylation of PI3K and Akt. Meanwhile, the levels of p53, Bax, and Bcl-2 were up-regulated. Thus, the occurrence and development of ESCC can be inhibited *in vivo* and *in vitro* (68). Not only that, CYH33, a novel selective PI3Kα inhibitor, showed a strong inhibitory effect on ESCC. The combination of CYH33 and IR can effectively inhibit tumor growth *in vivo* and *in vitro*. This was because co-treatment with CyH33 and IR further increased the levels of cleaved caspase 3 and γH2AX. Moreover, the quantity of G2/M phase cells were also increased. Hence, accelerating cell apoptosis and DNA damage. Notably, constitutively activated Akt disrupted the synergistic interaction between CyH33 and IR (69, 70)

### 5.3 mTOR Inhibitors

Rapamycin, an mTORC1 inhibitor, is the first mTOR inhibitor discovered in humans. Rapamycin has been shown to have inhibitory effects on a variety of cancers. It has the flexibility to work either alone or in combination with other drugs. For example, one study has demonstrated that both rapamycin and cisplatin alone can significantly suppress tumor growth, but the combination of them has the strongest anti-cancer effect. At the same time, rapamycin can also inhibit DNA synthesis, thus slowing the progression of cancers (71). It is worth noting that everolimus is an analogue of rapamycin, which has similar chemical properties to rapamycin. In one study, everolimus reduced phosphorylation of p70S6K and 4E-BP1 in TE4 cells with the highest p-mTOR content and TE11 cells with the lowest p-mTOR content. Moreover, in a mouse subcutaneous xenograft model, the combination of everolimus and cisplatin was found to have an additive effect on tumor growth inhibition, similar to rapamycin (72, 73). Temsirolimus is a selective mTOR inhibitor that is essentially a novel analog of rapamycin. In a mouse model of subcutaneous xenograft, temsirolimus has been shown to significantly reduce subcutaneous tumor growth in a dose-dependent manner (74). Moreover, several mTOR kinase inhibitors had inhibitory effects both on mTORC1 and mTORC2, and these drugs were known as dual mTORC1 and mTORC2 inhibitors, such as PP242. One study showed that both PP242 and rapamycin can affect the proliferation and cell cycle arrest of Eca-109 and TE-1 cell lines, but the efficiency of PP242 was higher than rapamycin. Furthermore, compared with rapamycin, PP242 inhibited the phosphorylation of Akt (S473) and p70S6K (T389), while rapamycin acted only on the latter. Additionally, PP242 had a synergistic effect with cisplatin, and PP242 could increase the apoptosis induced by cisplatin (75).

### 5.4 PI3K/Akt/mTOR Inhibitors and Drug Resistance

ESCC is a kind of cancer with a high degree of malignancy. Although there are many available drugs that can inhibit ESCC, the efficacy, toxicity and prognosis of drugs are not ideal. This is largely due to the existence of multiple resistance mechanisms that reduce the sensitivity of drugs to cancer. How to overcome drug resistance is a difficult point in cancer treatment. Studies have shown that monotherapy can often induce resistance through compensatory activation of downstream molecules. However, simultaneous targeting of multiple targets in the same signaling pathway may overcome this compensation. For example, the above mentioned PI3K inhibitor, LY294002, can inhibit the occurrence and development of ESCC by inhibiting the PI3K/Akt/mTOR/p70S6K signaling pathway. Nevertheless, compensatory activation of Akt Ser473 and PRAS40 at Thr246 might limit the inhibitory effect of LY294002 on ESCC cells, leading to resistance of ESCC to LY294002. In addition, they found that knockdown of rictor inhibited LY294002-induced Akt compensatory activation and reduced its resistance in ESCC cells. Thus, the anti-proliferation, metastasis and clone formation ability of LY294002 were improved (67). The dual mTORC1 and mTORC2 inhibitor PP242 had a similar effect, which was considered to be a sensitizer of cisplatin. In ESCC, PP242 can inhibit mTORC1 and mTORC2 pathways and regulate the constitutive activation of Akt induced by cisplatin, thus enhancing the anti-tumor effect of chemotherapy drug cisplatin. Ultimately, the sensitivity of ESCC cells to cisplatin chemotherapy was enhanced (75). Additionally, Moshe Elkabets et al. observed the levels of EGFR and S6 phosphorylation were increased in BYL719 resistant cells. At the same time, they demonstrated that BYL719, a specific PI3Kα inhibitor, was resistant through activation of mTOR activity (76). The underlying mechanism was as follows: AXL was a membrane-bound receptor tyrosine kinase, which was the most highly expressed gene in genomic analysis of drug-resistant cells. It can activate and phosphorylate EGFR in a ligand-independent manner. Furthermore, it caused the activation of PLCγ and PKC, which in turn led to the activation of mTOR independent of PI3K/Akt. In addition, they demonstrated that the combined inhibition of PI3Kα, EGFR, and PKC was far more effective on ESCC cells than monotherapy (76, 77). Researchers also found the resistance to rapamycin in ESCC patients, the reason is that rapamycin induced a large number of negative feedback loops from p70S6K to PI3K or mTORC2, which significantly activated the PI3K/Akt signaling pathway and weakened the anticancer effect of rapamycin (71, 75). OP16, a derivative of a novel NT-kaurene diterpene isolated from rubescens, significantly inhibited rapamycin-activated PI3K and reversed rapamycin-reduced rictor phosphorylation. Therefore, combined inhibition of PI3K and mTORC2 may be another way to circumvent the rapamycin-induced feedback loop (78).

## 6 PI3K/Akt/mTOR Inhibitors in Clinical Studies

Multiple PI3K/Akt/mTOR inhibitors have been shown to be effective *in vitro* and *in vivo* in ESCC. However, they are not yet used in clinical practice for ESCC treatment. Here we introduce the PI3K/Akt/mTOR inhibitors which have been under clinical evaluation in other gastrointestinal cancers, including gastric cancer (GC) and colorectal cancer (CRC). A lot of patients have received complete response (CR) or partial response (PR) under the PI3K/Akt/mTOR inhibitors treatment, providing important indications and possibilities for ESCC therapy (**Supplementary Table S2**).

### 6.1 Akt Inhibitors

**Capivasertib** (AZD5363) is a novel inhibitor of Akt. AZD5363 in combination with paclitaxel has been utilized in a phase II clinical trial in patients with PIK3CA mutation and PIK3CA amplification in advanced gastric adenocarcinoma.

**MK-2206**, an allosteric Akt inhibitor, has been tested in several clinical trials and showed good outcomes. These include advanced GC and esophagogastric junction cancers, as well as previously treated metastatic or locally advanced colorectal cancer that cannot be surgically removed. Stable disease (SD) was observed in 20% of GC and esophagogastric junction cancer patients treated with MK-2206, and the rate of radiation PR was 10%. Moreover, MK-2206 can also be used in combination with selumetinib in CRC. However, due to its low efficiency in targeting p-Akt and p-ERK, and high toxicity, these led to various adverse events (AEs), such as acneiform rash, blurred vision, nausea, etc. (79, 80).

**GDC-0068** is a selective Akt inhibitor. GDC-0068 combined with paclitaxel was found to improve PFS in the intent-to-treat (ITT) population in metastatic triple-negative breast cancer. The researchers compared the efficacy of GDC-0068 with placebo in combination with 5-fluorouracil, calcium folinolate, and oxaliplatin in advanced metastatic gastric cancer (MGC) and gastroesophageal junction cancer. They observed median PFS of 7.5 months in the placebo group and 6.6 months in the GDC-0068 group. This suggested that GDC-0068 did not improve PFS in GC patients who were not selected or biomarker selected (81).

### 6.2 PI3K Inhibitors

**BKM120** (Buparlisib) is a pan-class I PI3K inhibitor. It has been reported to inhibit the growth of a variety of cancers, particularly in PIK3CA mutant and KRAS wild-type tumor cells. Currently, BKM120 has been tested in phase II clinical trials in CRC patients with PIK3CA-activated mutations. In addition, three clinical trials of BKM120 in combination with mFOLFOX6, panitumumab or irinotecan in patients with metastatic colorectal cancer (MCRC) or advanced CRC have been completed. In a phase I clinical trial, the combination of BKM120 and mFOLFOX6 was shown to have a maximum tolerated dose (MTD) of 40mg/day, significantly lower than the 100mg/day alone. Due to the lack of targeting action of MTD at 40mg/day, the combination is not recommended (82, 83).

**BYL719** (Alpelisib) is a selective oral inhibitor of PI3Kα. In the phase II clinical trial of BRAF mutation MCRC, the overall response rate (ORR) and PFS in the combined treatment with BYL719, LGX818, and cetuximab were 18% and 4.2 months, respectively. But this is also accompanied by many AEs such as fatigue, vomiting, diarrhea, dermatologic AEs (rashes, dermatitis acneiform, dry skin, melanocytic nevus) and hyperglycemia. At present, this study has been completed, but the efficacy of BYL719 still needs further experimental evaluation (84).

**PX-866** is a specific PI3K inhibitor. Currently, the clinical trial of PX-866 combined with cetuximab in incurable MCRC has entered the phase I clinical trial and demonstrated stunning anticancer effects. In the 9 patients evaluated for efficacy, both the patients with PR and SD accounted for 44.4% (85).

### 6.3 mTOR Inhibitors

**Temsirolimus** (CCI-779) is an inhibitor of mTOR that has been extensively studied in different clinical trials. It is currently approved for the treatment of advanced renal cell carcinoma, but its treatment for CRC is still in clinical trials. The combination of temsirolimus and irinotecan in MCRC patients with KRAS mutations has entered phase II clinical trials and a significant increase in the proportion of patients with SD and reduced tumors was observed (86).

**Everolimus** has shown strong anticancer activity in many cancers. The safety and efficacy of everolimus plus best supportive care (BSC) in patients with advanced GC has entered phase III clinical trial. They found a tendency to reduce the risk of death with everolimus and found that the estimated median survival with everolimus combined with BSC was 5.4 months, compared with 4.3 months in the placebo group. Moreover, everolimus has often been studied in combination with other drugs, such as bevacizumab, irinotecan, cetuximab, mFOLFOX-6, OSI-906, AV-951 and panitumumab, which have been extensively studied in MCRC, RMCRC, and advanced CRC, while mitomycin C and capecitabine have been primarily studied in GC (87–98).

## 7 Conclusion

The PI3K/Akt/mTOR pathway is one of the most complex regulatory networks in the human body, and the abnormality of main components stimulated the occurrence of cancer. Currently, most studies have focused only on a few common forms of aberrations, such as PIK3CA, PTEN, Akt1, and Akt2. Abnormal changes in these genes have provided potential targets for cancer treatment. Therefore, in order to explore more effective therapeutic approaches, it is necessary to investigate other aberrations about this pathway. At the same time, in order to achieve the desired clinical benefit, we also need to understand the various molecules that regulate the oncogenic function of this pathway. These molecules can directly or indirectly regulate the PI3K/Akt/mTOR pathway through a variety of ways, thereby affecting the proliferation, metastasis, chemoradiotherapy sensitivity and angiogenesis of ESCC.

Several PI3K/Akt/mTOR pathway inhibitors have been investigated to be effective against a variety of cancers, and sufficient clinical data have been obtained. For example, the pan-PI3K inhibitor buparisib (BKM120) has shown antitumor activity in estrogen receptor (ER) positive breast cancer and xenograft tumors, either alone or in combination, and has been studied in phase III clinical trials in breast cancer. Similarly, everolimus has shown a strong antitumor effect in advanced HER2-positive breast cancer and advanced GC, which have also progressed to phase III clinical trials (93, 99). Unfortunately, while many targeted inhibitors of ESCC (such as MK226 and everolimus) have been discovered, no drugs have been approved for clinical use due to severe side effects and drug resistance. Because monotherapy often leads to compensatory activation of other pathways, such as LYZ294002, rapamycin, and cisplatin.LYZ294002 is a pan-PI3K inhibitor that induces compensatory activation of Akt during its inhibitory action, thereby reducing its inhibitory effect. Similarly, BYL719 is a specific PI3K inhibitor that has been shown to have inhibitory effects in head and neck squamous cell carcinoma. BYL719 has also been found to be effective in ESCC but its efficacy is often limited by drug resistance (76). Dual inhibitors can effectively reduce compensatory activations and enhance therapeutic effects. Therefore, in order to overcome this redundant pathway activation, new drugs or multi-drug combinations should be vigorously developed. Therefore, future research should focus on the study of other forms of mutations, the exploration and discovery of new regulatory molecules, and the combination therapy or the development of dual inhibitors to overcome the resistance problems caused by monotherapy.

PI3K/Akt/mTOR pathway is one of the most vital pathway regulating the basic physiological functions of cells. Abnormal activation of this pathway are usually caused by the regulation of its upstream molecules and mutations or amplification of major components (e.g. PIK3CA, Akt1, PTEN, etc.). Although many inhibitors have been shown to be effective in PI3K/Akt/mTOR signaling pathway, clinical studies are still lacking. Moreover, drug resistance has been a persistent problem. The efficacy of a multi-drug combination is superior to that of medication alone in preventing the compensatory activation of other pathways. Therefore, the research focus should be on multi-drug combination therapy and search for multi-inhibitors.

## Author Contributions

All listed authors made significant and intellectual contribution to this work. XL designs this review. ZD provides guidance. QL wrote the text and collected related data. RD and WL provided assistance on the outline and language. GH is responsible for revising the content of the article. All authors contributed to the article and approved the submitted version.

## Funding

This work was supported by National Natural Science Foundation of China (No. 82172996, 82073075); Science and technology project of Henan Province (No. 182102310324, 202102310206); Training plan for young backbone teachers of Zhengzhou University (No. 2018ZDGGJS037); Training plan for young backbone teachers of Henan Province (No. 2020GGJS010); Basic research and Cultivation Fund for young teachers of Zhengzhou University (No. JC202035023); Science and technology innovation talents support plan of Henan Province (No.21HASTIT048); Innovation team support plan for outstanding young talents of Zhengzhou University.

## Conflict of Interest

The authors declare that the research was conducted in the absence of any commercial or financial relationships that could be construed as a potential conflict of interest.

The reviewer HG declared a shared parent affiliation with the authors to the handling editor at the time of review.

## Publisher’s Note

All claims expressed in this article are solely those of the authors and do not necessarily represent those of their affiliated organizations, or those of the publisher, the editors and the reviewers. Any product that may be evaluated in this article, or claim that may be made by its manufacturer, is not guaranteed or endorsed by the publisher.
