# Supplementary material for: PI3K/Akt/mTOR Signaling Pathway: Role in Esophageal Squamous Cell Carcinoma, Regulatory Mechanisms and Opportunities for Targeted Therapy
Source: Front Oncol. 2022 Mar 22;12:852383. doi: 10.3389/fonc.2022.852383 (PMC8980269; doi:10.3389/fonc.2022.852383)
Supplement: Supplementary file 1 [file Table_1.docx]

**Supplementary material**

**Supplementary Table S1.** PI3K/Akt/mTOR pathway inhibitors in ESCC.

| **Name** | **Structure** | **Animal mode** | **concentriation** | **Efficacy** |
| --- | --- | --- | --- | --- |
| Oridonin | 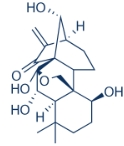 | mouse (SCID) model | 40 or 160 mg/kg orally, once a day for 52 days | Tumor growth in 160 mg/kg Cordonidin group was reduced by about 50% compared with the control group. |
| Xanthohumol | 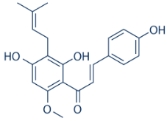 | Patient-derived xenograft mouse (SCID) mode | 80 or 160 mg/kg orally for 50 consecutive days | Compared with the vehicle-treated group, in HEG5 cases, the inhibition rate of 160 mg/kg xanthohumol group was 30%, and EG9 or HEG18 could reach 50%. |
| scutellarin | 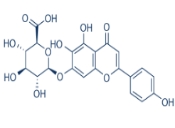 | Patient-derived xenograft mouse (SCID) mode | 50 or 100 mg/kg were gavaged continuously for 16 days | Tumor volume decreased by more than 50% compared to the vehicle-treated group. |
| triciribine (TCN) | 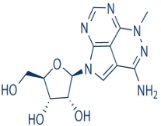 | ECA109 cells were subcutaneously inoculated into male BALB/c nude mice | They were divided into control group, 25 mg/kg TCN group, 4 Gy ionizing radiation (IR) group, 25 mg/kg TCN + IR group (6Gy), each group was treated for 24 days | In the radiotherapy group injected with TCN, the doubling time was significantly prolonged to 13.8±0.8 days after combined treatment, which enhanced the inhibitory effect of radiation on ESCC. |
| MK2206 | 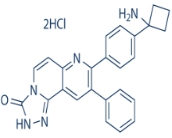 | KYSE 150 cells were subcutaneously inoculated in male nude mice | MK2206 (90 mg/kg), BEZ235 (15 mg/kg), MK2206 (30 mg/kg) combined with BEZ235 (5 mg/kg) were given intragastrically 3 times a week for 2 weeks | MK2206 alone had only a slight inhibitory effect on tumor growth. However, MK2206 and BEZ235 significantly inhibited tumor growth compared to the control group at lower doses. |
| BEZ235 | 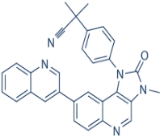 | KYSE 150 cells were subcutaneously inoculated in male nude mice | BEZ235 (15 mg/kg), MK2206 (30 mg/kg) combined with BEZ235 (5 mg/kg) were intraperitoneally injected 3 times a week for 2 weeks | The combination of BEZ235 and MK2206 had a significant therapeutic effect compared with that of BEZ235 alone |
| LY294002 | 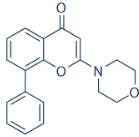 | ECA109-rictor shRNA or ECA109-control shRNA cells were subcutaneously inoculated in nude mice | The dose of 5 mg/kg was gavaged every two days | Compared with the untreated group, LY294002 treatment significantly inhibited tumor growth |
| AZD8186 | 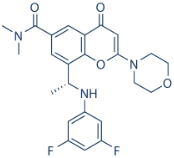 | KYSE70 cells were injected subcutaneously into nude mice | 5mg/kg, 10mg/kg AZD8186 and DTX were injected by caudal vein every 3 days for 21 days, except for free AZD818 | AZD8186 had a mild antitumor effect, but the combination with docetaxel significantly induced tumor regression or stagnation |
| CYH33 | 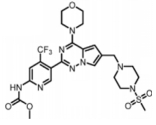 | The patient-derived tumor tissue was allografted into BALB/ Ca mice | CYH33 (12.5 mg/kg, once a day), 2Gy radiation (day 0, 1, 7,8, 9, 28) | The T/C value of CYH33 was 35.49%, and the radiation of 2 Gy was 61.19%. CYH33 and IR significantly improved the curative effect, and the T/C value was 25.93%. |
| Rapamycin | 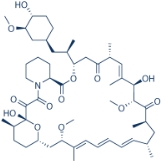 | EC9706 cells were subcutaneously into athymic BALB/c nude mice | 50μg/kg rapamycin, 1 mg/kg cisplatin  were injected twice a day for 2 weeks. | Compared with the control group, the inhibition rates of rapamycin, cisplatin and rapamycin combined with cisplatin were 41.04%, 27.13% and 80.78%, respectively. |
| everolimus | 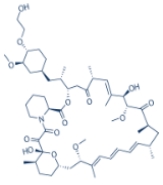 | Subcutaneous xenograft of TE4 cells and TE11 cells in nude mice | Everolimus 5mg/kg was given twice a week and cisplatin 3mg/kg every two weeks | Tumor load in mice treated with everolimus and cisplatin was reduced by 83% and 68%, respectively, compared with placebo, and the combination of the two drugs reduced the tumor load by 92% |
| Temsirolimus | 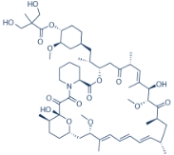 | The xenograft model of TE-8 cells in BALB/cA nude mice | Temsirolimus is given weekly at a dose of 10 mg/kg | Median survival (control group, 31 days;Temsirolimus group, 43 days;P = 0.0024)  Tumor volume in mice (control group, 3261.6±722.0;Temsirolimus group, 599.2±122.9;P = 0.007) |
| PP242 | 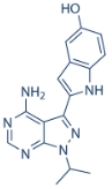 | NA | 1 nmol/ L to 10 μ mol/ L PP242 and rapamycin were used for MTT assay, while 0.5 and 1 μmol/ L PP242 and rapamycin were used for colony formation assay | The cell inhibition rate of rapamycin was 40-60%, and PP242 at higher concentration could inhibit more than 80% cell growth, which was more effective than rapamycin. |

**Supplementary Table S2.** Summary of PI3K/ Akt/mTOR Inhibitors in Clinical Trials

| **Drug name** | **Combination** | | **Patients** | | **phase** | **Clinical Trial ID** | | **Administration** | | **Efficiency** | | **Ref** | |  |
| --- | --- | --- | --- | --- | --- | --- | --- | --- | --- | --- | --- | --- | --- | --- |
| Akt Inhibitors |  | |  | |  |  | |  | |  | |  | |  |
| AZD5363 | Paclitaxel | | Advanced GC patients harboring PIK3CA mutation and / or PIK3CA amplification (n=21) | | II | NCT02451956 | | AZD5363 400mg bid 4 days/3 days off of a 7 day cycle, weekly. Paclitaxel 80mg/m^2^, days 1, 8 and 15 of a 28 day cycle. | | NA | | NA | |  |
| MK-2206 | Single agent | | Advanced GC or gastroesophageal junction cancer (n=75) | | II | NCT01260701 | | 60mg MK-2206, orally, every other day. | | The response rate was 1%; the median progression-free survival was 1.8 months and the median OS was 5.1 months. | | (79) | |  |
|  | Single agent | | RMCRC (n=18) | | II | NCT01802320 | | MK2206 PO on days 1, 8, 15 and 22, repeated every 28 days. | | NA | | NA | |  |
|  | Selumetinib | | Advanced CRC (n=21) | | II | NCT01333475 | | MK-2206 90mg/135mg, weekly; Selumetinib 75mg/100mg, daily. | | 1 patient had SD. | | (80) | |  |
| GDC-0068 | 5-Fluorouracil   leucovorin  oxaliplatin | | GC (n=153) | | II | NCT01896531 | | 600mg GDC-0068, PQ, QD, on days 1-7 of 14-day cycle.  Oxaliplatin 85mg/m^2^, T_IV_> 2 h, on day 1 of 14-day cycle.  Leucovorin 400mg/m^2^. 5-FU, 400/2400mg/m^2^, T_IV_ =46–48 h. | | Median PFS was 6.6 months; 37 patients had ORR; 2 patients had CR; 35 patients had PR; 20 patients had SD. | | (81) | |  |
| PI3K Inhibitors | |  | |  | | |  | |  | |  |  |  | |
| BKM120 | Single agent | | CRC with PIK3CA activating mutations | | II | NCT01501604 | | 100mg orally (PQ), once daily (QD). | | NA | | NA | |  |
|  | mFOLFOX6 | | MCRC (n=17) | | I | NCT01571024 | | BKM120 40mg, orally, once daily. mFOLFOX6 was administered every 2 weeks. | | 3 patients had SD; 4 patients had PD at the time of their first evaluation. | | (82) | |  |
|  | Panitumumab | | Metastatic/Advanced RAS-wild type CRC (n=22) | | I–II | NCT01591421 | | 6mg/kg panitumumab, IV, every 14 days. 60mg BKM120, PQ, QD. | | 7 patients had SD; 8 patients had PD. | | (83) | |  |
|  | Irinotecan | | Advanced CRC (n=20) | | I | NCT01304602 | | BKM120, PQ, QD. Irinotecan: T_IV_> 90 minutes, every 2 weeks. | | NA | | NA | |  |
| BYL719 | LGX818  cetuximab | | BRAF mutant MCRC (n=156) | | II | NCT01719380 | | 200mg LGX818, 300mg BYL719 and cetuximab, daily. | | 5 patients had PR and 21 patients had SD. PFS was 4.2 months. | | (84) | |  |
| PX-866 | Cetuximab | | Incurable MCRC (n=178) | | I–II | NCT01252628 | | PX-866, 6-8mg, QD.  Cetuximab, 400mg/m^2^ (followed by 250mg/m^2^), weekly. | | 4 patients had PR, 4 patients had SD and 1patients had PD. | | (85) | |  |
| mTOR Inhibitors | |  | |  | | |  | |  | |  |  |  | |
| Temsirolimus | Irinotecan | | MCRC (n=50) | | II | NCT00827684 | | Temsirolimus 25mg, IV. Irinotecan 180mg/m^2^, IV, combine with temsirolimus (15mg ~ 25mg) once every 2 weeks. | | Temsirolimus: median TTP= 45days; 38% patients had SD.  Temsirolimus+ irinotecan: median TTP= 84days; 63 % patients had SD. | | (86) | |  |
| Everolimus | Single agent | | MCRC (n=199) | | II | NCT00419159 | | Everolimus 10mg/day or 70mg/week. | | 70mg/week group: DCR was 31%; 25 patients had SD, 58 patients had PD.  10mg/day group: DCR was 32.4%; 26 patients had SD, 55 patients had PD. | | NA | |  |
|  | Cetuximab  irinotecan | | MCRC (n=100) | | II | NCT01387880 | | 1.66mg per day up to 7½ mg per day | | NA | | NA | |  |
|  | Bevacizumab | | MCRC (n=50) | | II | NCT00597506 | | 10mg everolimus daily, orally.  10mg/kg IV bevacizumab given days 1 and 15 of each cycle. | | 8 patients had minor responses (16%), 15 patients had SD (30%). Median PFS and OS times were 2.3months and 8.1 months. | | (87) | |  |
|  | Irinotecan  cetuximab | | MCRC (n=19) | | I | NCT00478634 | | Everolimus 30mg/week, orally; irinotecan 400 or 250mg/m^2^, loads IV, weekly. Cetuximab 350 or 250mg/m^2^ every 3 weeks. | | Dose level A1: median PFS and OS were 2.9 months and 5.8 months, respectively. Dose level B1: median PFS and OS were 5.9 months and 17.5 months, respectively. | | (88) | |  |
|  | Cetuximab | | MCRC or recurrent colon cancer (n=12) | | I | NCT01637194 | | Everolimus PO QD on days -14 and then 1-28; cetuximab T_IV_> 60-120 minutes on days -7 and then once weekly. | | NA | | NA | |  |
|  | FOLFOX  bevacizumab | | CRC (n=47) | | I–II | NCT01047293 | | 5mg/kg bevacizumab; mFOLFOX-6 (oxaliplatin 85mg/m^2^, folinic acid 400mg/m^2^, 5-fluorouracil 400mg/m^2^), IV, on day 1 and 15 of every cycle. Everolimus (dose level 1-3: 5-10mg), PO. | | 3 patients had CR, 22 patients had a PR; ORR was 53% in the intent-to-treat group and 64% in the evaluable group. | | (89) | |  |
|  | OSI-906 | | RMCRC (n=18) | | I | NCT01154335 | | OSI-906, 50mg/100mg, BID.  Everolimus 5mg/10mg, QD. | | 4 patients had SD at the first evaluation; the median OS was 30.6 weeks. | | (90) | |  |
|  | AV-951 | | RMCRC (n=56) | | I–II | NCT01058655 | | Phase I, PO, QD.  Cohort 1: everolimus 5mg + AV-951 1mg. Cohort 2: everolimus 10mg + AV-951 1mg. Cohort 3: everolimus 10mg + AV-951 1.5mg.  Phase II, PO, QD.  everolimus 10mg + AV-951 1mg. | | Median PFS and OS times were 3.0 months and 5.6 months. The 2-month PFS rate was 50%; 20 patients had SD. | | (91) | |  |
|  | Irinotecan  panitumumab | | Advanced CRC (n=49) | | I–II | NCT01139138 | | Panitumumab 6mg/kg IV every 14 days; irinotecan 200mg/m^2^ IV every 14 days; everolimus 5/10mg, PO, QD. | | RR was 48% and SD was 43%; median PFS was 5.6 months, and the median OS was 11.8 months. In RAS/RAF WT patients, RR was 60%, median PFS was 6.4 months, and OS of 11.8 months. | | (92) | |  |
|  | Single agent | | Advanced GC (n=656) | | III | NCT00879333 | | Everolimus 10mg (2 tablets x 5mg), PO, QD. | | Median PFS was 1.7 months, and the median OS was 5.4 months. | | (93) | |  |
|  | Capecitabine | | RGC (n=59) | | I–II | NCT01099527 | | Capecitabine 650mg/m^2^ twice daily (Day1-14) and everolimus 5mg twice daily (Day1-21) every 3 weeks. | | 5 patients had PR, 18 patients had SD; ORR= 10.6%, disease control rate= 48.9%; median PFS= 11 weeks, median OS= 21 weeks. | | (94) | |  |
|  | Single agent | | Advanced GC (n=45) | | II | NCT01482299 | | Everolimus 10mg, PO, QD. | | NA | | NA | |  |
|  | MitomycinC | | Advanced GC, cancer of the esophagogastric junction (n=16) | | I | NCT01042782 | | RAD001 5mg, 7.5mg or 10mg, PO, QD (3cohorts); mitomycin C 5mg/m^2^ IV every 3 weeks. | | 3 patients had PR, 4 patients had SD. | | (95) | |  |
|  | Single agent | | Advanced GC (n=54) | | II | NCT00519324 | | Everolimus 10mg, PO, QD. | | The DCR was 56.0%; median PFS was 2.7 months, and the median OS was 10.1 months. | | (96) | |  |
|  | Cisplatin  5-FU  leucovorin | | Advanced GC (n=40) | | II | NCT00632268 | | Everolimus: oral 10mg/day on Day 1,8,15.  Cisplatin: infusion 35mg/m^2^/day on Day 1, 8. 5-FU: infusion 2000mg/m^2^/day on Day 1,8,15. | | 21 patients had PR; the ORR was 52.5%; median PFS was 6.9 months, and the median OS was 10.5 months. | | (97) | |  |
|  | Single agent | | Unresectable or metastatic esophageal cancer or stomach cancer (n=49) | | II | NCT00985192 | | Everolimus 10mg, PO, QD. | | 2 % had PR, 39% had SD; median PFS was 1.8 months, and the median OS was 3.4 months. | | (98) | |  |

Progressive Disease (PD); Complete Response (CR); Time Progress(TTP); Disease control rate (DCR); Refractory colorectal cancer(RCRC); Refractory metastatic colorectal cancer(RMCRC); Refractory gastric cancer(RGC).
